# Cardiovascular Surveillance in BRAF/MEK Inhibitor Therapy: Early Lessons from Prospective Data

**DOI:** 10.1016/j.jaccao.2025.09.003

**Published:** 2025-11-14

**Authors:** Viraj Shah, Tarek Nahle, Avirup Guha

**Affiliations:** a Division of Cardiology, Department of Medicine, Medical College of Georgia at Augusta University, Augusta, Georgia, USA; b Cardio-Oncology Program, Medical College of Georgia at Augusta University, Augusta, Georgia, USA

**Keywords:** biomarkers, BRAF/MEK inhibitors, CTRCD, echocardiography, reversibility

**Figure:**
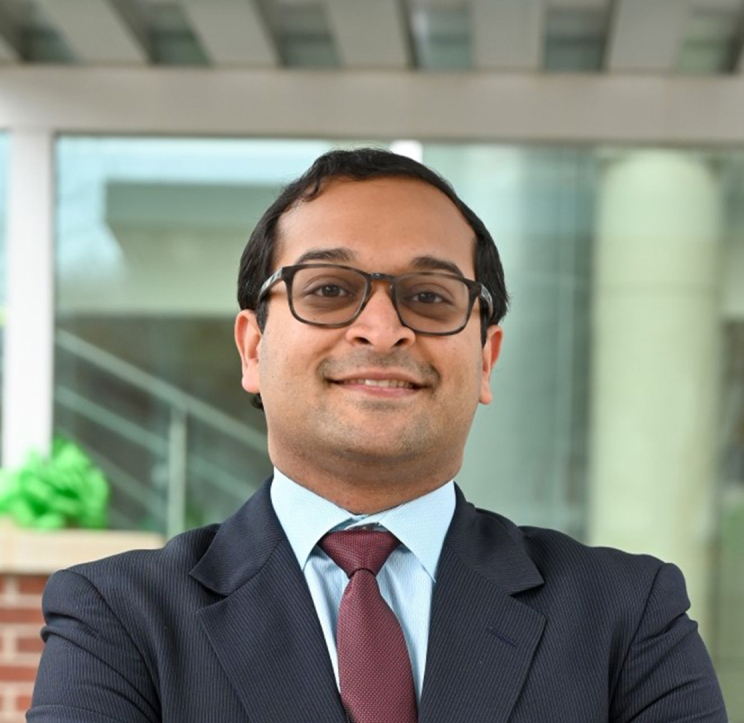

Over the past decade, BRAF and MEK inhibitors have transformed outcomes for patients with BRAF-mutant melanoma and are increasingly applied in non–small cell lung and colorectal cancer. These agents substantially improve survival, yet, as with many cancer therapies, come at a cost of cardiovascular adverse events, including hypertension, left ventricular (LV) systolic dysfunction, arrhythmia, and QT prolongation.^1^ Clinical trials have signaled these risks but often underestimated them due to short follow-up, reliance on Common Terminology Criteria for Adverse Events (CTCAE) grading, and exclusion of patients with baseline cardiovascular disease.^2^, ^3^, ^4^, ^5^, ^6^ Glen et al^7^ now provide the first prospective, multimodality study specifically designed to evaluate the cardiovascular effects of BRAF/MEK inhibitors in advanced melanoma.

In this 61-patient cohort followed for 24 weeks, nearly one-half (46%) developed cancer therapy–related cardiac dysfunction (CTRCD), predominantly mild. Importantly, 6.6% experienced moderate or severe dysfunction, all within the 4 weeks of therapy initiation. All moderate/severe cases were managed with temporary interruption of therapy and initiation of guideline-directed heart failure therapy (angiotensin-converting enzyme inhibitor/angiotensin receptor blocker [ACE-I/ARB] and beta-blocker), with successful rechallenge once LV ejection fraction improved. No recurrent severe dysfunction occurred after rechallenge. Elevated baseline N-terminal pro–B-type natriuretic peptide (NT-proBNP) was associated with dysfunction, whereas serial biomarkers had limited longitudinal yield. Notably, no low-risk patient by Heart Failure Association–International Cardio-Oncology Society (HFA–ICOS) criteria developed moderate/severe dysfunction.^8^ Strengths include systematic echocardiography, biomarkers, home blood pressure monitoring, cardiac magnetic resonance in select cases, and consistent application of IC-OS definitions. Limitations are modest size, an all-White population, and short, 6-month follow-up precluding assessment of late outcomes such as symptomatic heart failure or arrhythmia.

The 2022 ESC Cardio-Oncology Guidelines recommend baseline HFA–ICOS assessment, echocardiography at 4 weeks, and 3-monthly imaging thereafter.^9^ Glen et al^7^ support a more pragmatic, risk-adapted strategy: concentrate imaging early, de-escalate once stability is achieved, rely on home blood pressure (BP) monitoring instead of frequent clinic visits, and limit biomarker testing mainly to baseline.

Contextualizing within prior evidence (Table 1), clinical trials have reported LV systolic dysfunction in 2% to 12% of patients, limited by selective enrollment and inconsistent definitions.^2^, ^3^, ^4^, ^5^, ^6^ Retrospective registries (MarketScan, FAERS [U.S. Food and Drug Administration Adverse Event Reporting System]) and Danish cohorts suggested higher cardiovascular adverse event rates of 8% to 27%, with 2% to 14% major dysfunction, mostly reversible.^10^, ^11^, ^12^, ^13^ Glen et al’s prospective study^7^ clarifies these discrepancies: CTRCD is common, but typically mild; moderate/severe events are uncommon, arise early, and are reversible with guideline-directed therapy. Hypertension emerges consistently across all data sets, with an incidence of 30% to 50% when structured monitoring is employed.^7^ One fatal venous thromboembolism (VTE) also occurred, echoing registry and trial signals (coBRIM [A Study Comparing Vemurafenib Versus Vemurafenib Plus Cobimetinib in Participants With Metastatic Melanoma], COLUMBUS [Study Comparing Combination of LGX818 Plus MEK162 Versus Vemurafenib and LGX818 Monotherapy in BRAF Mutant Melanoma]).^2^^,^^3^^,^^10^^,^^11^ Yet VTE screening is absent from European Society of Cardiology and National Comprehensive Cancer Network guidelines, reflecting a blind spot in cardio-oncology practice.^9^^,^^14^

**Table 1:** Registry, Observational, and Prospective Studies of BRAF/MEK Inhibitor–Associated Cardiotoxicity

Study Name	Study Details	HTN, %	CTRCD/LVSD, %	Arrhythmia, %	VTE, %	Reversibility
Pivotal trials^2^, ^3^, ^4^, ^5^, ^6^	Data source: phase III melanoma trials	3+ (6%-26%)	2+ (2%-12%)	1+ (Arr/QTc 2%-5%)	1+ (3%-5%)	–
	Population/surveillance quality: low-risk; only capture CTCAE- (registration intent)					
Guha 2021 (FAERS + MarketScan)^10^	N/data source: FAERS: 7,752 reports (4,659 combination therapy)/MS: 657 reports (112 combination therapy)	2+ (FAERS 1.3%; MS 11.1%)	1+ (FAERS 2.4%; MS 4.8%)	1+ (FAERS 1.3%; MS 2.1%)	1+ (FAERS 2.6%; MS 6.2%)	–
	Population/surveillance quality: mixed comorbidity; PV + claims					
Barbieri 2025 (FAERS)^11^	N/data source: 18,370 reports on BRAF/MEK in FAERS; 1,591 (8.7%) cardiac adverse events	–	1+ 4.0%	1+ 2.6%	1+ 4.5%	–
	Population/surveillance quality: global FAERS; PV only					
Pedersen (2022)^12^	N/data source: 139 melanoma Denmark	–	2+ (Major 11%)	–	–	(80%)
	Population/surveillance quality: observational; serial MUGA					
Oddershede (2025)^13^	N/data source: 170 melanoma Denmark	–	2+ (Major 14%)	2+ (AF 12%)	–	(∼79%)
	Population/surveillance quality: observational; pragmatic echo/MUGA					
Glen (Retrospective, 2023)^8^	N/data source: 63 UK	–	2+ (CTRCD 27%; 10% mod)	–	–	(Reversibility suggested)
	Population/surveillance: retrospective; ICOS echo definitions					
Glen (Prospective, 2025)^7^	N/data source: 61 UK	4+ (46%)	1+ (6.6% mod/sev)	–	VTE (1 fatal PE)	(All mod/sev reversible; ∼40% mild recovered)
	Population/surveillance: prospective; echo 4 wk + biomarkers + BP + CMR					

Percentages or percentage ranges (%) represent either: 1) incidence estimates when derived from clinical trials, insurance claims, or observational cohorts; or 2) proportions of adverse event reports when derived from pharmacovigilance (PV) databases. Signal strength grading (1+ to 4+) and corresponding color shading are based directly on % thresholds: 1+ (<10%, light yellow), 2+ (10%-20%, pale orange), 3+ (20%-30%, orange), 4+ (≥30%, red-orange). Reversibility % (green) indicates the proportion of events that improved with intervention when reported.

– = not observed or not reported. AF = atrial fibrillation; Arr = arrhythmia; BP = blood pressure; CM = cardiomyopathy; CMR = cardiac magnetic resonance; CTCAE = Common Terminology Criteria for Adverse Events; CTRCD = cancer therapy–related cardiac dysfunction; FAERS = U.S. Food and Drug Administration Adverse Event Reporting System; HF = heart failure; HTN = hypertension; ICOS = International Cardio-Oncology Society; LVSD = left ventricular systolic dysfunction; mod = moderate; MS = MarketScan claims database; MUGA = multigated acquisition scan; PE = pulmonary embolism; sev = severe; VTE = venous thromboembolism.

Management of CTRCD is evolving toward a hold–treat–rechallenge approach. ESC guidelines endorse withholding therapy for moderate/severe dysfunction, continuing therapy in mild cases with close monitoring, and considering early initiation of ACE-I/ARB and beta-blockers. Mechanistic rationale exists for beta-blockers in MEK inhibitor–induced dysfunction via suppression of p38 MAPK signaling.^9^ Data from registry analyses (eg, Oddershede et al^13^) also support reversibility in ∼80% of cases, reinforcing the importance of early detection and intervention.^12^

The study, therefore, delivers several practice-changing insights for cardio-oncology practice.^7^ First, timing matters: all clinically significant dysfunction occurred within the first month, making early, front-loaded surveillance critical, particularly prioritizing a 4-week echocardiogram in medium- and high-risk patients only. Second, risk stratification is effective: HFA–ICOS categories, supplemented by baseline NT-proBNP, reliably identified those most vulnerable to dysfunction, providing a simple and scalable strategy.^8^ Third, BP monitoring is essential: home BP checks detected more hypertension than clinic visits, underscoring their role in structured surveillance. Fourth, management is feasible: a hold–treat–rechallenge paradigm enabled safe continuation of oncologic therapy without recurrent severe dysfunction. Finally, although nearly one-half of patients developed CTRCD, most cases were mild, did not progress to more severe dysfunction over 6 months, and were reversible in 70% to 80% of cases with timely intervention.

Future directions include the following: Larger multicenter prospective registries linking oncologic and cardiovascular outcomes; mechanistic studies to explain interpatient variability in dysfunction; integration of biomarkers, imaging phenotyping, and digital tools (eg, home BP, wearables); preventive trials testing cardioprotective agents such as ACE-I/ARB, beta-blockers, or SGLT2 inhibitors; evaluating the necessity of long-term surveillance in low-risk or stable patients to avoid over-monitoring; and considering VTE surveillance or prophylaxis as part of cardio-oncology practice with BRAF/MEK inhibitors (Table 1).

## Funding Support and Author Disclosures

Dr Guha is supported by American Heart Association-Strategically Focused Research Network Grant in Disparities in Cardio-Oncology (#847740, #863620) and Department of Defense Prostate Cancer Research Program’s Physician Research Award (#HT94252310158). The authors have reported that they have no relationships relevant to the contents of this paper to disclose.
